# Importance of the Rhizosphere Microbiota in Iron Biofortification of Plants

**DOI:** 10.3389/fpls.2021.744445

**Published:** 2021-12-03

**Authors:** Tristan Lurthy, Barbara Pivato, Philippe Lemanceau, Sylvie Mazurier

**Affiliations:** Agroécologie, AgroSup Dijon, INRAE, University of Bourgogne, University of Bourgogne Franche-Comté, Dijon, France

**Keywords:** iron, biotic interactions, plant–microbe interaction, microbiota, plant nutrition, iron biofortification, rhizosphere

## Abstract

Increasing the iron content of plant products and iron assimilability represents a major issue for human nutrition and health. This is also a major challenge because iron is not readily available for plants in most cultivated soils despite its abundance in the Earth’s crust. Iron biofortification is defined as the enhancement of the iron content in edible parts of plants. This biofortification aims to reach the objectives defined by world organizations for human nutrition and health while being environment friendly. A series of options has been proposed to enhance plant iron uptake and fight against hidden hunger, but they all show limitations. The present review addresses the potential of soil microorganisms to promote plant iron nutrition. Increasing knowledge on the plant microbiota and plant-microbe interactions related to the iron dynamics has highlighted a considerable contribution of microorganisms to plant iron uptake and homeostasis. The present overview of the state of the art sheds light on plant iron uptake and homeostasis, and on the contribution of plant-microorganism (plant-microbe and plant-plant-microbe) interactions to plant nutritition. It highlights the effects of microorganisms on the plant iron status and on the co-occurring mechanisms, and shows how this knowledge may be valued through genetic and agronomic approaches. We propose a change of paradigm based on a more holistic approach gathering plant and microbial traits mediating iron uptake. Then, we present the possible applications in plant breeding, based on plant traits mediating plant-microbe interactions involved in plant iron uptake and physiology.

## Introduction

More than 820 million people are suffering from chronic undernourishment, and two billion from micronutrient deficiencies (hidden hunger) worldwide (FAO et al., 2019). People from lower-middle-income countries suffer from hunger (undernourishment) and do not have access to a varied diet (malnutrition). This is leading to micronutrient deficiencies (MNDs) in micronutrients such as iron (Fe), Zinc (Zn) and vitamin A. Overcoming undernourishment and overcoming malnutrition represent two of the main “Sustainable Development Goals” of the United Nations Development Program UNDP 2015 (UNDP, 2015).

Iron deficiency—the main case of MND in the world—has serious effects on human health, such as microcytic anemia, impaired immune function and poor endocrine function (Bailey et al., 2015; Wakeel et al., 2018; World Health Organization, 2021). Iron plays an essential role in the physiology of living organisms, e.g., in DNA synthesis, respiration, and photosynthesis (Aisen et al., 2001).

Meat is the main source of iron in food, with 20–60% of Fe in the form of haemoproteins that are easily assimilable by the human body (Cross et al., 2012). However, a significant fraction of the world human population does not have access to meat and thus suffers from iron deficiency. This deficiency also occurs in developed countries, especially among young ladies (Beck et al., 2014). In plant-based diets in developing countries, iron is supplied by plant products, especially grains (cereals and legumes). However, these grains contain low levels of Fe, and even more include anti-nutritional compounds such as phytates which hamper Fe assimilation (Gómez-Galera et al., 2010). Yet, the iron concentration in grains has decreased over the years because of intensified agriculture, e.g., with the introduction of semi-dwarf, high-yielding cultivars of wheat (Fan et al., 2008). At the same time, meat consumption is decreasing in developed countries with the growing concern for environmental (higher environmental footprint of animal proteins than of plant proteins) and diet issues. Thus, increasing Fe content and assimilability in plant products represents a major challenge for human nutrition and health.

To address this challenge, food fortification, which consists in artificially supplementing food with micronutrients (iron, zinc, vitamins), has been proposed as an option. The Food Fortification Initiative^1^ created a network of governmental and private agencies in several developing countries to promote the iron fortification of wheat flour. As a result, the consumption of Fe-fortified wheat flour has increased from 18% in 2004 to 27% in 2007, allievating iron deficiency for 540 million people (White and Broadley, 2009). Promising results were also obtained in India, the Philippines and Rwanda with rice, pearl millet and beans supplemented with iron (Finkelstein et al., 2017, 2019). However, there are limitations to this approach. Costs are important, supplementation may modify food taste and is not always well accepted, and finally fortified food hardly reaches poor people with limited or no access to commercial channels. Iron fertilization is a common agricultural practice also used to mitigate plant iron deficiency but not considered so far overlooked to increase staple food quality. Three main groups of Fe fertilizers are used: inorganic Fe compounds, synthetic Fe, and organic Fe complexes (Abadía et al., 2011; Zanin et al., 2019). In addition to their high cost, the possible incorporation of these ligands into edible parts of the plant (Abadía et al., 2011) may represent a problem. These limitations also apply to new nano-chelates under development (Yuan et al., 2018). Indeed, the increasing use of nanoparticles raises concerns for human health or the environment (Soares and Soares, 2021). In short, efficient Fe fertilizers have several drawbacks: they are expensive, their efficiency is variable, and they can be incorporated in the host plant including its edible parts. They do not represent sustainable options for increasing the iron content of agricultural products, even if foliar applications of iron may be of interest in specific cases (e.g., increasing the iron content of rice and barley grains, Slamet-Loedin et al., 2015).

Soil microorganisms have long been known to contribute to plant iron nutrition (Marschner, 1995). This beneficial effect was first showed by comparing the iron contents of plants grown in sterile and non-sterile soils. The iron content of sunflower, maize (Masalha et al., 2000), rape and red clover (Rroço et al., 2003; Jin et al., 2006) was significantly lower when they were grown in sterile rather than non-sterile soil; sunflower even suffered from chlorosis in sterile soil. A high occurrence of oligotrophic bacteria in lupine rhizosphere was associated with an increased concentration of Fe, Cu, Mn and Zn in plant shoots, suggesting that these bacteria may contribute to plant iron and more generally to plant mineral nutrition (De Santiago et al., 2019). The promotion of iron nutrition in a range of plant species by various microbial strains (e.g., root symbionts) and metabolites has been reported in a series of studies listed in Table 1. Interactions between plants may also facilitate their iron nutrition. Intercropping cereal and legume plants can notably improve their iron content (Zuo et al., 2000; Gunes et al., 2007; Zuo and Zhang, 2009; Xue et al., 2016). Thus, maize-peanut intecropping improved Fe nutrition of peanut (Zuo et al., 2000), while wheat-chickpea intecropping increased the Fe concentration in wheat seeds (Gunes et al., 2007). Interestingly, the rhizosphere microbiota of these associated plant species differed from the rhizosphere microbiota of these same plants cultivated separately (Sun et al., 2009; Zhang et al., 2012; Wahbi et al., 2016a; Taschen et al., 2017). A more complex rhizosphere bacterial network was recently shown in pea-wheat intecropping (Pivato et al., 2021). Thus, we can hypothesize that the rhizosphere microbiota accounts for the increased iron uptake by intercropped plants.

**TABLE 1:** Microorganisms and microbial metabolites mediating the plant iron status.

Microorganisms and/or microbial metabolites	Application modes	Plants	Effects on the plant iron status	Additional observations	Effects on plant genes	Mechanism(s) proposed by authors	References
*Acinetobacter calcoaceticus* O-13; *Bacillus simplex* K-10	Bacterial suspension	Potato	Plant [Fe]/Fe^(1)↗(2)^	Tryptophan addition enhance iron uptake		Sid.^(3)^ iron mobilization	Mushtaq et al., 2021
N_2_ fixer and/or auxin producer mutants of *Azospirillum brasilence* FP2	Bacterial suspension	Maize	Plant [Fe]/Fe↗, modif. Fe distrib^(3)^.	Root ethylene production↘^(4)^, root auxin and DIMBOA^(5)^ production↗, metabolic partitioning of carbon differed		Regulation of hormone signaling and cellular iron transport	Housh et al., 2021
*Gluconacetobacter diazotrophicus* PAL5; *Azospirillum brasilense* REC3	Bacterial suspension	Strawberry	Plant [Fe]/Fe↗	Phenolic compounds content↘, chlorophyll↗		Sid. iron mobilization	Delaporte-Quintana et al., 2020
*Pseudomonas* spp.; *Enterobacter* spp.; *Bacillus sporothernodurans*	Bacterial suspension	Sunflower	Plant [Fe]/Fe↗	Sid.^(6)^ production↗, phytohormone production↗, phosphate solubilization↗, HCN^(7)^ production↗		Sid. iron mobilization	Pourbabaee et al., 2018
*Burkholderia cepacia* JFW16	Bacterial suspension	Milkvetch	Plant [Fe]/Fe↗	Rhizosphere acidification, root FR^(8)^↗, flavin release, sid. and phytohormone production↗	*FRO2* expr.^(9)^↗, *IRT1* expr.↗, *AHA2* expr.↗, *FIT1* expr.↗	Promotion of iron mobilization by acidification, strategy I iron uptake, and hormonal regulation	Zhou et al., 2018
*Pseudomonas fluorescens* ATCC13525	Bacterial suspension	Tomato	Plant [Fe]/Fe↗		*IRT1* expr.↗, *FRO2* expr.↗, *NRAMP3* expr.↗	Promotion of strategy I iron uptake, and redistribution	Nagata, 2017
*Burkholderia terricola* LMG20594; *Pseudomonas brassicacearum* NFM421; *B. pyrrocinia* LMG14191; *P. mandelii* NBRC103147; *Herbaspirillum huttiense* NBRC10252	Bacterial suspension	Lentil, pea	Plant [Fe]/Fe↗	Rhizosphere acidification, sid. production↗, phytohormone production↗		Iron uptake	Reza, 2017
*Paenibacillus polymyxa* BFKC01	Bacterial suspension	Arabidopsis	Plant [Fe]/Fe↗	Root FR↗	*FRO2* expr.↗, *IRT1* expr.↗, *FIT1* expr.↗ MYB72 expr.↗	Promotion of iron uptake by modulation of the expression of strategy I key genes and of ISR key genes	Zhou et al., 2016
*Rhizobium leguminosarum* bv.^(10)^ *phaseoli*; *Pseudomonas* spp. Avm	Bacterial suspension	Common bean	Plant [Fe]/Fe↗, modif. Fe distrib.	Wild variety more efficient in Fe uptake than cultivated variety after microbial inoculation		Promotion of iron uptake	Carrillo-Castañeda et al., 2005
*Bacillus subtilis* CPA; *Bacillus* sp. AHP3; *Pseudomonas chlororaphis* PR29; *Glomus fasciculatum* (consortium)	Bacterial and fungal suspension	Wheat	Plant [Fe]/Fe↗	Grain protein content↗superoxide dismutase (SOD)↗catalase (CAT)↘, chlorophyll↗Metabolome modification		Promotion of nutrient yield by metabolic regulation and ROS scavenging activity	Yadav et al., 2020
*Arthrobacter sulfonivorans* DS-68; *Enterococcus hirae* DS-163	Bacterial coating	Wheat	Plant [Fe]/Fe↗, seed [Fe]/Fe↗, modif. Fe assimil.^(11)^	Anti-nutritional factor↘		Promotion of iron uptake	Singh et al., 2018
*Bacillus subtilis* BHHU10, *Trichoderma harzianum* TNHU27, and *Pseudomonas aeruginosa* PJHU15 (consortium)	Bacterial and fungal coating	Pea	Modif. Fe assimil.	Phenolics, flavonoids, ascorbic acid and protein content↗		Promotion of ROS scavenging activity in plants	Jain et al., 2014
*Pseudomonas fluorescens* C7R12; pyoverdine of *P. fluorescens* C7R12	Bacterial suspension; apo-siderophore	*Arabidopsis*	Root [Fe]/Fe↘, shoot [Fe]/Fe↗	Changes in plant hormone production, Incorporation of Fe-pyoverdine suggested by ^15^N-labeling and immunodetection	Numerous modifications evidenced in a transcriptomic study	Sid. promotion of iron mobilization in the rhizosphere including the apoplast, of strategy I iron uptake, and regulation of hormone signaling	Trapet et al., 2016
*B. subtilis* GBO3	Bacterial suspension; Bacterial VOCs^(12)^	*Arabidopsis*	Plant [Fe]/Fe↗	Rhizosphere acidification, root FR↗	*FRO2* expr.↗, *IRT1* expr.↗, *FIT1* expr.↗	Promotion of iron mobilization by acidification and of strategy I iron uptake	Zhang et al., 2009
*Bacillus amyloliquefaciens* BF06	Bacterial VOCs	*Arabidopsis*	Plant [Fe]/Fe↗	Root FR↗, Fe^2+^ production↗, Production of VOCs implied (2R or 3R-butanediol)	*FRO2* expr.↗, *IRT1* expr.↗, *FIT1* expr.↗	Promotion of strategy I iron uptake through gene expression modulation	Wang et al., 2017
*Arthrobacter sulfonivorans* DS-68; Arthrobacter sp. DS-179	Liquid bacterial culture coating	Wheat	Plant [Fe]/Fe↗	Organic acid production↗	*ZIP* expr.↗	Promotion of iron uptake and translocation through organic acid production and stimulation of iron transporters	Singh et al., 2017
*B. subtilis* GBO3	Liquid bacterial culture	Cassava	Shoot [Fe]/Fe↗			Promotion of the plant iron status through the regulation of the plant iron metabolism including hormone signaling	Freitas et al., 2015
*Paenibacillus cookie* JGR8; *Pseudomonas pseudoalcaligenes* JGR2; *Bacillus megaterium* JGR9	Liquid bacterial culture	Lesser bullrush	Shoot [Fe]/Fe↗for strain JGR2, modif. Fe distrib.	Sid. production↗, phytohormone production↗, phosphate solubilization↗		Sid. promotion of iron accumulation and translocation; relationship between sid. production and phosphate solubilization	Ghosh et al., 2014
*Chryseobacterium* spp. C138	Liquid bacterial culture	Tomato	Plant [Fe]/Fe↗			Fe-sid. used as a source of iron under iron deficiency	Radzki et al., 2013
*P. putida* MTCC 103, Enterobacteria	Liquid bacterial culture	Rice	Plant [Fe]/Fe↗, seed [Fe]/Fe↗	Variation of peroxidase activity		Promotion of iron solubilization, uptake and translocation related to sid. production	Sharma et al., 2013
*R. leguminosarum* PR1; *Pseudomonas* sp. PGERs17	Liquid bacterial culture	Lentil	Plant [Fe]/Fe↗	Nodulation↗, leghaemoglobin↗		Fe-sid. used as a source of iron under iron deficiency	Mishra et al., 2011
*Trichoderma asperellum* T34	Fungal conidia	Cucumber	Shoot [Fe]/Fe↗			Fe-sid. used as a source of iron under iron deficiency	De Santiago et al., 2013
*T. asperellum* T34	Fungal conidia	White lupin	Shoot [Fe]/Fe↗	Peroxidase activity↗, catalase activity↗		Sid. promotion of iron accumulation and translocation under iron deficiency; promotion of ROS scavenging activity is implied	De Santiago et al., 2009
*Hymenoscyphus ericae*	Fungal suspension	Heather	Plant [Fe]/Fe↗	Variation in results depending on calcium addition		Sid. iron mobilization	Leake et al., 1990
*Glomus etunicatum* WV579A, *G. diaphanum* WV579B, *G. intraradices* WV894	Fungal cultures	Maize	Root [Fe]/Fe↗	Variation in results depending on soil pH and fungal strain		Sid. iron mobilization	Clark and Zeto, 1996
*Glomus mossae* and rhizosphere microorganisms	Fungal spores, root pieces and soil	Peanut, sorghum	Plant [Fe]/Fe↗modif. Fe distrib.	Plant phosphate↗		Increased soil exploration	Caris et al., 1998
*Glomus mossae, G. albidum, G. fasciculatum, G. macrocarpum.*	Fungal spores propagated in sterile soil	Galleta grass	Plant [Fe]/Fe↗	use of ^59^Fe		Sid. iron mobilization and transport into mycorrhizal plants	Cress et al., 1986
*Glomus intraradices*	Commercial inoculant	Maize	Shoot [Fe]/Fe↗Shoot [Fe]/Fe↘	Variation in results with amount of micronutrients and P added		Increased soil exploration	Liu et al., 2000
Arbuscular mycorrhiza fungi inoculant	Commercial inoculant	Chickpea	Plant [Fe]/Fe↗	No effect of mineral N fertilization		Increased soil exploration	Farzaneh et al., 2011
*Glomus intraradices, G. mosseae, G. aggregatum, G. etunicatum*	Commercial inoculant	Sorghum	Plant [Fe]/Fe↗	Plant biomass↗, chlorophyll↗, Plant S↗, ROS↘	*DMAS2* exp.↗, *NAS2* exp.↗, *YS1* exp.,↗	Promotion of strategy II iron uptake (PS↗), and of ROS scavenging activity	Prity et al., 2020
*Glomus intraradices, G. mosseae, G. aggregatum, G. etunicatum*	Fungal spores (mix)	Alfalfa	Plant [Fe]/Fe↗	Plant biomass↗, chlorophyll↗, plant S↗, root FR↗, ROS↘	*FRO* expr.↗, *SULTR* (*1;1, 1;2,1;3, 3;1)* expr.↗	Promotion of iron mobilization in the rhizosphere including the root apoplast, and of ROS scavenging activity	Rahman et al., 2020
*Glomus intraradices, G. mosseae, G. aggregatum, G. etunicatum*	Fungal spores (mix)	Sunflower	Plant [Fe]/Fe↗	Plant biomass↗, chlorophyll↗, root FR↗, ROS↘, CAT↗, SOD↗	*FRO1* expr.↗, *IRT1* expr.↗, *ZIP1* expr.↗,	Promotion of iron mobilization and uptake, and ROS scavenging activity	Kabir et al., 2020
*Rhizophagus irregularis* DAOM197198	Fungal spores	Maize	Shoot [Fe]/Fe↗	Genes implied in strategy II were not induced	*OPT8* expr.↗, *NAS* expr.↗	Selective induction of putative iron transporters	Kobae et al., 2014
*Rhizophagus irregularis D*AOM197198	Fungal spores	Chicory	Root [Fe]/Fe↗	Root exploration volume↗, phosphatase production↗		Increased soil exploration, phosphatase activities implied	Labidi et al., 2012
Desferrioxamine B, sid. of *Streptomyces* obtained commercially	Fe-siderophore	Wheat	Plant [Fe]/Fe↘, modif. Fe distrib.	Variation of phytosiderophore production		Sid. inhibition of iron uptake via PS chelation	Sadrarhami et al., 2021
3 pyoverdines, sids of *P. fluorescens* C7R12; *Pseudomonas* sp. B4214; *Pseudomonas* sp. D426	Fe-siderophore	Pea	Plant [Fe]/Fe↗, modif. Fe distrib.	Effects on the plant Fe status varying with pea cv.^(13)^ and sids, modifications of the plant ionome		Fe-sid. used as a source of iron under iron deficiency	Lurthy et al., 2020
Azotochelin, sid. of *Azotobacter vinelandii* obtained commercially	Fe-siderophore	Soybean	Plant [Fe]/Fe↗			Sid. iron mobilization	Ferreira et al., 2019
Pyoverdine, sid. of *P. fluorescens* ATCC13525	Fe-siderophore	Tomato	Plant [Fe]/Fe↗,	chlorophyll↗	*FRO2* expr.↗, *IRT1* expr.↗	Fe-sid. used as a source of iron under iron deficiency	Nagata et al., 2013
Pyoverdine, sid. of *P. fluorescens* C7R12	Fe-siderophore	*Arabidopsis*, tobacco, barley, wheat, fescue, rye grass	Plant [Fe]/Fe↗	Incorporation of Fe-pyoverdine suggested by ^15^N-labeling		Fe-sid. used as a source of iron under iron deficiency	Shirley et al., 2011
*Pseudomonas* spp. sid.	Fe-siderophore	Red clover	Plant [Fe]/Fe↗	chlorophyll↗		Sid. iron mobilization, Fe-sid. used as a source of iron under iron deficiency	Jin et al., 2010
Pyoverdine, sid. of *P. fluorescens* C7R12	Fe-siderophore	*Arabidopsis*	Plant [Fe]/Fe↗	An *IRT*1 mutant still incorporated Fe-pyoverdine, incorporation of Fe-pvd suggested by ^15^N-labeling and immunodetection		Fe-sid. used as a source of iron under iron deficiency using a non-reductive uptake mechanism	Vansuyt et al., 2007
Aerobactin, sid. of *Citrobacter diversus*	Fe-siderophore	Soybean	Plant [Fe]/Fe↗	Fe^2+^ production↘		Fe-sid. used as a source of iron under iron deficiency using a non-reductive uptake mechanism	Chen et al., 2000
Hydroxamate, sid. mixture from *Penicillium chrysogenum*	Fe-siderophore	Cucumber, maize	Plant [Fe]/Fe↗	Fe^2+^ production↗		Sid. iron mobilization	Hördt et al., 2000
Rhizoferrin, sid. of *Rhizopus arrhizus*	Fe-siderophore	Tomato	Modif. Fe distrib., root [Fe]/Fe↗	chlorophyll↗		Sid. iron mobilization	Yehuda et al., 2000
Ferrioxamine B, sid. of *Streptomyces* spp. obtained commercially	Fe-siderophore	Onion	Root [Fe]/Fe↗	Root FR unchanged		Sid. iron mobilization	Manthey et al., 1996
Rhizoferrin, sid. of *Rhizopus arrhizus*	Fe-siderophore	Tomato	Modif. Fe distrib., root [Fe]/Fe↗			Fe-sid. used as a source of iron under iron deficiency	Shenker et al., 1995
Pseudobactin, syn.^(14)^ pyoverdine, sid. of *Pseudomonas putida* WCS358	Fe-siderophore	Barley	Modif. Fe distrib., root [Fe]/Fe↗	No Fe exchange between pyoverdine and phytosiderophore		Fe-sid. used as a source of iron under iron deficiency	Duijff et al., 1994
Ferrioxamine B, sid. of *Streptomyces* spp. obtained commercially	Fe-siderophore	Cucumber	Plant [Fe]/Fe↗	Siderophore in the xylem		Fe-sid. used as a source of iron, uptake through the transpiration stream and translocation	Wang et al., 1993
Ferrioxamine B, sid. of *Streptomyces* spp. obtained commercially	Fe-siderophore	Cotton, maize	Root [Fe]/Fe↗	Fe removal from chelate around the root, chlorophyll↗		Sid.-mediated iron uptake	Bar-Ness et al., 1992
Ferrioxamine B, sid. of *Streptomyces* spp. obtained commercially	Fe-siderophore	Pine	Plant [Fe]/Fe↘ in mycorrhizal plant			Sid.-mediated iron uptake	Leyval and Reid, 1991
Ferrichrome A, sid. of *Ustilago sphaerogena* ATCC 12421; ferrioxiamine B, sid. of *U. sphaerogena* ATCC 12421	Fe-siderophore	Oat	Plant [Fe]/Fe↗			Fe-sid. used as a source of iron under iron deficiency using a specific sid. uptake mechanism	Crowley et al., 1988
Agrobactin, sid. of *Agrobacterium tumefaciens* B6	Fe-siderophore	Pea, bean	Shoot [Fe]/Fe↗	Chlorophyll↗		Fe-sid. used as a source of iron	Becker et al., 1985a
Pseudobactin, syn. pyoverdine, sid. of *Pseudomonas aureofaciens* (now commonly referred to as *Pseudomonas chlororaphis*) ATCC15926	Fe-siderophore	Pea, maize	Shoot [Fe]/Fe↘	Chlorophyll↘		Sid. competitive bidding of iron	Becker et al., 1985b

*^(1)^[Fe]/Fe, iron concentration and/or iron amount; ^(2)^↗, increase; ^(3)^Modif. Fe distrib., modified Fe distribution; ^(4)^↘, decrease; ^(5)^DIMBOA, (2,4-dihydroxy-7-methoxy-1,4-benzoxazin-3-one); ^(6)^Sid(s)., siderophore(s); ^(7)^HCN, hydrogen cyanide; ^(8)^FR, ferric reductase; ^(9)^expr., expression; ^(10)^bv., biovar; ^(11)^Modif. Fe assimil., modified Fe assimilability; ^(12)^VOC(s), volatile organic compound(s); ^(13)^cv., cultivar; ^(14)^syn., synonym.*

On the basis of a range of studies published lately, we argue that iron biofortification is a relevant option to alleviate MND. This option requires better knowledge of the organisms and mechanisms that promote plant iron uptake and homeostasis. The present overview of the state of the art sheds light on plant iron uptake and homeostasis, and on the plant-microorganisms interactions (plant-microbe and plant-plant-microbe) that impact these processes. Then, we describe different strategies of iron fortification of plants, with a special focus on biofortication, and we finally discuss promising prospects based on the monitoring of the dynamic interplay between plants and their rhizosphere microbiota, including microbes from the surrounding soil, attached to and influenced by the roots, plus from the roots themselves (endophytes).

## Biological Levers to Promote Plant Iron Uptake and Regulate Iron Homeostasis

### Valuing Plant Genetic Resources to Improve Iron Nutrition

#### Plant Iron Physiology

**FIGURE 1:**
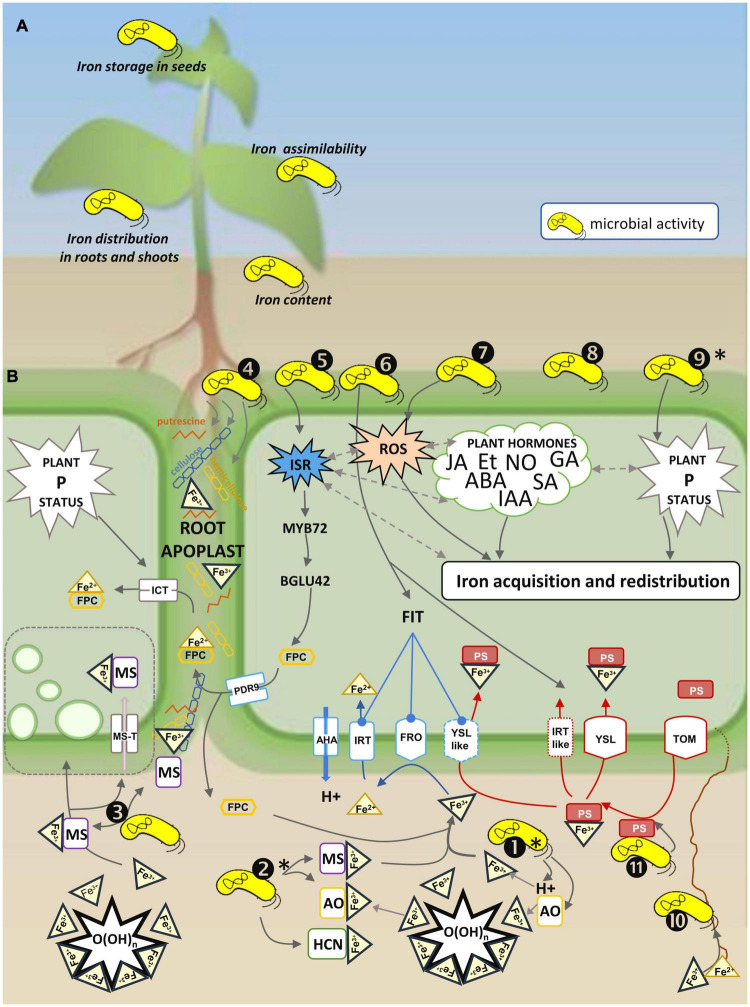
Schematic representation of different aspects of microbial regulation of the plant iron status. **(A)** Types of effects of microorganisms on the plant iron status: (i) content, (ii) root and/or shoot distribution, (iii) assimilability, and (iv) iron storage in the seeds (see Table 1 for further information). **(B)** Microbial activities involved in the active strategy of plant iron uptake and homeostasis. Plant transporters and enzymes mediating iron uptake are represented in blue for dicots and non-graminaceous monocots, and in red for grasses. In iron uptake strategy I (reductive strategy), protons are extruded by H^+^-ATPases (e.g., AHA in *Arabidopsis*), Fe^3+^ is reduced by plant ferric reductases (e.g., FRO2 in *Arabidopsis*) to Fe^2+^ which is internalized in root cells by a specific transporter (e.g., IRT1 in *Arabidopsis* or IRT-like in rice and barley). Excretion of root fluorescent phenolic compounds (FPC) *via* ABC transporters (e.g., PDR9 in *Arabidopsis*) contributes to strategy I iron uptake and more specifically to re-mobilization of root apoplastic iron which is internalized via IRT1. In iron uptake strategy II (chelating strategy), Fe^3+^ is chelated by phytosiderophores (PS) (e.g., secreted via TOM1 in rice) and the Fe-PS complex is internalized by a specific transporter (e.g., YSL in rice or YSL-like in peanut). A non-reductive mechanism controlled by the plant phosphorus (P) status implies a putative specific iron chelate transporter (ICT) possibly involved in the internalization of chelates formed by Fe^3+^ reduced and complexed by FPC (Fe^2+^-FPC). Numbers represent the types of microbial activities: ➊Acidification through production of protons (H^+^), organic acids (OA) and hydrogen cyanide (HCN), and ➋Chelation by OA, HCN or microbial siderophores (MS) contributes to solubilization of ferric iron (Fe^3+^) immobilized in O(OH)n. Acidification and chelation increase bioavailability of Fe^3+^ to plants. ➌Fe^3+^-MS complexes are suspected to be internalized by a non-reductive process (MS transport, MS-T) with possible endocytosis (represented by green vesicles). ➍Rhizosphere microorganisms can metabolize cellulose, hemicellulose and putrescine, or produce putrescine, and thus modify molecules involved in root apoplastic iron storage and re-mobilization. ➎Microorganisms triggering induced systemic plant resistance (ISR) influence FPC production; FPCs contribute to plant iron uptake by re-mobilizing root apoplastic iron through the modulation of *MYB72* (root transcription factor) and B*GLU42* (beta-glucosidase) expression. ➏Microorganisms influence the expression of key genes of plant iron uptake strategies I and II. ➐Microorganisms influence the plant reactive oxygen species (ROS) status, and this modulates the plant iron status. ➑Microorganisms influence the plant hormone (JA, jasmonic acid; Et, ethylene; ABA, abscisic acid; NO, nitric oxide; GA, gibberellin; SA, salicylic acid; IAA, indole acetic acid) status (directly by synthesizing or metabolizing them, or indirectly by inducing plant defense responses), and this modulates the plant iron status. ➒Microorganisms influence the plant P status, which is implied in the regulation of the plant iron status. ➓Fungal root symbionts extend the volume of soil explored, and this improves acquisition of nutrients including iron. ⓫Phytosiderophore scavenging by soil bacteria influences strategy II plant iron uptake. Slash-dotted arrows indicate interrelations between various components of plant physiology influencing plant iron acquisition and redistribution. (*) influence on plant P bioavailability.

The forms of iron available to plants are the ferric iron cation (Fe^3+^), or ferric-ion chelates (Fe^3+^-chelates), and the ferrous iron cation (Fe^2+^) (Figure 1). Two main strategies of root iron acquisition are described: strategy I (the reduction-based strategy), and strategy II (the chelation strategy) (Curie and Briat, 2003; Curie et al., 2009; Kobayashi and Nishizawa, 2012; Connorton et al., 2017). Strategy I is found in non-graminaceous monocots and dicots. It relies on the reduction of Fe^3+^ by a ferric reduction oxidase (encoded by a *FRO* gene), and the incorporation of the resulting Fe^2+^ into the root by an iron-regulated transporter (encoded by an *IRT* or a *RIT* gene). The pH is decreased in the rhizosphere (Hinsinger et al., 2003), as a result of proton extrusion by plasma membrane proton pumps (encoded by an *AHA* gene); this acidification increases Fe^3+^ solubility.

Strategy II is found in grasses. It relies on the excretion of phytosiderophores (PSs, structural derivatives of mugineic acid) by a transporter of mucigenic acid (encoded by a *TOM* gene) that chelates Fe^3+^ (Fe^3+^-PS) before incorporation into the root by an oligopeptide transporter belonging to the Yellow Stripe/Yellow Stripe Like family (YS/YSL) (Curie et al., 2009).

Differentiating plant species according to their iron uptake strategy has its own limitations, as both strategies are found in rice, barley and peanut (Ishimaru et al., 2006; Pedas et al., 2008; Xiong et al., 2013). In *Arabidopsis* (a strategy I plant), chelating agents (e.g., coumarins) may contribute to iron nutrition in addition to the reduction strategy, especially in alkaline environments (Fourcroy et al., 2014; Schmid et al., 2014; Schmidt et al., 2014). These fluorescent phenolic compounds (FPCs) are synthesized *via* the phenylpropanoid pathway and secreted *via* an ABC transporter (e.g., PDR9 in *Arabidopsis*). Chlorotic phenotypes of *IRT*1 and *FRO*2 mutants were not restored by FPCs from plant exudates, suggesting that strategy I is implied in the uptake of Fe^3+^chelated to PC (Fourcroy et al., 2016). However, based on a review of results obtained under phosphate- (Pi-) deficient conditions, Tsai and Schmidt (2017) hypothesized the existence of an auxiliary IRT1-independent iron chelate transporter (ICT) that would bypass the IRT1 uptake system and internalize the Fe-FPC complex under Pi-deficient conditions. Flavins, another family of phenolic compounds, also increased iron solubilization in the rhizosphere of other plant species (e.g., barrel medic, sugar beet) than *Arabidopsis* (Rodríguez-Celma et al., 2013; Sisó-Terraza et al., 2016). Iron solubilization by phenolic compounds has also been reported in rice, a strategy II plant (Bashir et al., 2010; Ishimaru et al., 2011). Besides PSs and FPCs, organic acids (e.g., citrate or succinate) chelate Fe^3+^ (Fe^3+^-OC) and contribute to plant iron nutrition (Abadía et al., 2011; Adeleke et al., 2017). Synthetic ferric chelates (Fe^3+^-SC)—e.g., Fe-EDDHA or Fe-EDTA –, may provide iron to strategy I plants; entire chelates have been found in roots and shoots (Orera et al., 2009, 2010). These small hydrophilic molecules are suspected to use the transpiration stream as the driving force of entry (Abadía et al., 2011). Iron uptake by the leaves follows similar mechanisms as those described in the roots (Malhotra et al., 2019). Thus, even if strategies I and II remain essential pathways for iron acquisition by plants, they are not necessarily exclusive and may be complemented by additional mechanisms.

Iron is a central cofactor of enzymatic reactions involving electron transfer in essential metabolic pathways such as respiration or photosynthesis (Balk and Schaedler, 2014). Yet, its redox properties may also induce toxic effects. Free Fe^2+^ catalyzes the Fenton reaction in which reactive oxygen species (ROS) characteristic of oxidative stress are generated, and ROS may cause irreparable damage to cellular components when they are present in excessive concentrations (Winterbourn, 1995). The intracellular concentrations and forms of iron are therefore tightly regulated at the cellular level. The mechanisms involved in iron homeostasis have many common features in strategy I and strategy II plants (Connorton et al., 2017; Grillet and Schmidt, 2019; Kobayashi et al., 2019). *In planta*, chelation of Fe^2+^ to Nicotianamine (NA), and Fe^3+^ to citrate, allows iron mobilization and the control of its high reactivity (Curie et al., 2009; Connorton et al., 2017). In strategy II plants, ferric iron is additionally present in the form of Fe^3+^-PS (Zhang et al., 2019). Iron is also stored in unreactive forms such as ferritins that represent a major iron pool in plants mostly found in chloroplasts and mitochondria. They behave like a buffer that stores iron to avoid overload and the resulting ROS formation, and releases it when needed. To avoid oxidative stress, iron storage in vacuoles also contributes to iron homeostasis. Candidate transporters for moving cytosolic iron into vacuoles are members of the IRT, FPN/IREG (ferroportin/iron regulated) and VIT (vacuolar iron transporter) families (Morrissey et al., 2009; Roschzttardtz et al., 2009; Vert et al., 2009). VIT transporters are particularly important for vacuolar iron storage in seeds (Roschzttardtz et al., 2009; Zhang et al., 2012). In seeds, iron is mainly found under an insoluble form poorly available for nutrition because it is complexed with phosphate bound to inositol in phytates (Mary et al., 2015). Iron remobilization from the vacuoles is mediated by members of the natural resistance-associated macro-phage protein family (NRAMP) (Curie et al., 2000; Nevo and Nelson, 2006). In addition to ferritins and vacuoles, the root apoplast appears as a third level of iron storage by plants (Curie and Mari, 2017). Bienfait et al. (1985) demonstrated that a pool of 500–1,000 nanomoles of Fe *per* gram fresh weight could be formed in the root apoplast where it is adsorbed on the cell wall whose net charge is negative (Shomer et al., 2003). The cell wall composition, more particularly the respective proportions of celluloses, hemicelluloses, pectins, and lignins, varies depending upon plant genotypes, and influences the amount of adsorbed iron (Chen, 2014; Shi et al., 2018). The plant capacity to store iron in its root apoplast is positively correlated to the amount of hemicelluloses in the cell wall and would be a key determinant in the IDC tolerance of graminaceous plant species such as maize (Shi et al., 2018). IDC tolerance in soybean is also associated with an increased amount of root apoplastic iron (Longnecker and Welch, 1990). Remobilization of precipitated apoplastic iron relies on phenolic compounds (Jin et al., 2007; Bashir et al., 2011; Ishimaru et al., 2011; Lei et al., 2014), putrescine synthesis, and decreased cell wall suberization (Zhu et al., 2016; Curie and Mari, 2017).

Proteins involved in iron acquisition—F6′H1, PDR9, and members of the HA, FRO, and IRT family—also mediate cellular iron trafficking (Connorton et al., 2017). Members of the oligopeptide transporter (OPT) protein family (e.g., YS/YSL transporters) are also key determinants of iron transport *in planta* (Su et al., 2018; Grillet and Schmidt, 2019; Kumar et al., 2019). This transporter family is particularly important for the transport of Fe to the seeds (Grillet et al., 2014; Curie and Mari, 2017; Su et al., 2018; Kumar et al., 2019).

#### Plant Breeding

The identification of plant traits mediating plant iron uptake and iron homeostasis *in planta* offers opportunities for plant breeders to promote iron nutrition and content in agricultural products (Waters and Sankaran, 2011).

Conventional plant selection and new breeding strategies are both applied to enhance iron acquisition, storage and nutritional availability in edible parts of crops. There exists a natural genetic variation in the level of expression of the mechanisms regulating iron uptake and homeostasis among plant genotypes. The efficiency of plant iron nutrition is highly variable across plant species (Hansen et al., 2006). The level of tolerance or the susceptibility to IDC highly differs according to plant species and even to cultivars (e.g., Gildersleeve and Ocumpaugh, 1989; Zribi and Gharsalli, 2002; Mahmoudi et al., 2009; Helms et al., 2010). The Fe levels in grains can vary significantly depending on cultivars (e.g., from 10 to 160 mg/kg in maize, 15–360 mg/kg in wheat, 23–105 mg/kg in pea and 34–157 mg/kg in bean) (White and Broadley, 2005, 2009). Assimilability of Fe for human beings correlates positively with the iron content in edible parts of crops (Welch et al., 2000) and varies with the forms of iron. The most assimilable forms of iron in plants are Fe^2+^-nicotianamine (Fe^2+^-NA) and Fe^3+^-ferritin (Zielińska-Dawidziak, 2015; Beasley et al., 2019). In addition, iron nutritional availability is decreased by antinutrient molecules (e.g., phytates and tannins) that hamper its assimilation. Sufficient iron availability in food products is only possible when the concentration of these molecules is low (Sandberg, 2002; Delimont et al., 2017). Conventional breeding has led to the selection of cultivars showing better resistance to iron stress (i.e., IDC tolerant) and a higher iron content in edible parts, but also a decreased content of antinutrients (reviewed in Garcia-Oliveira et al., 2018). This was the case in species displaying high natural variability in their iron content (e.g., bean and pearl millet) (Manwaring et al., 2016; Lockyer et al., 2018). The selection of IDC-tolerant cultivars has further improved yields under iron stress conditions. However, the corresponding selection process relies on a long and costly screening of inbred lines.

Taking that limitation into account, transgenesis has been proposed as an option to promote plant iron nutrition and content by overexpressing or silencing genes mediating plant iron acquisition, transport and/or storage. The corresponding strategy has been followed through the targeting of one gene or several ones in combination and has led to genotypes with an increased iron content (i.e., from <2 to 6-fold) (Kawakami and Bhullar, 2018; Connorton and Balk, 2019). However, transgenesis raises public concerns (Lassoued et al., 2018). Furthermore, positive effects recorded in controlled conditions may be lost in field conditions because iron bioavailability varies among soils (Gregory et al., 2017). For example, iron uptake by soybean was increased by overexpressing *FRO* in controlled iron stress conditions (Vasconcelos et al., 2006) but not in high-calcareous soil environments (Kocak, 2014); even more, this genetic transformation appeared to be deleterious under non-iron stressed conditions due to toxic effects of the iron overload (Vasconcelos et al., 2006).

More generally, results from cultivars obtained from conventional and new breeding strategies vary depending upon soil iron bioavailability (Gregory et al., 2017; Garcia-Oliveira et al., 2018; Lockyer et al., 2018; Connorton and Balk, 2019). Alternative strategies based on QTL (quantitative trait loci) identification and on genome-wide association (GWAS) have been proposed to identify putative traits and genes mediating plant iron nutrition, and include them in plant breeding programs. The first step of this strategy confirmed the importance of genes implied in (i) iron uptake strategies I and II, (ii) the synthesis of phenolic compounds, and (iii) iron homeostasis. They further underlined the multigenic character of traits related to the plant iron status and the crucial importance of environmental conditions (Garcia-Oliveira et al., 2018; Connorton and Balk, 2019). Gene expression profiling of soybean plants sensitive or tolerant to IDC pinpointed key roles for phenylpropanoids (Waters et al., 2018). The major contribution of iron storage in the root apoplast and of fluorescent phenolics to remobilize this extracytoplasmic iron was confirmed and represents potential breeding targets (Curie and Mari, 2017; Waters et al., 2018). The complex and interregulated mechanisms of plant iron uptake and homeostasis has also been emphasized. A key role has been given to (i) phosphorus known to be in close relation with the iron status (e.g., Vansuyt et al., 2003; Tsai and Schmidt, 2017; Shi et al., 2018; Filiz and Kurt, 2019), (ii) ISR through the root-specific transcription factor MYB72 and beta-glucosidase BGLU42 (Zamioudis et al., 2014), and (iii) hormone signaling, especially IAA, Et, NO and ABA signaling (Lei et al., 2014; Li et al., 2015; Curie and Mari, 2017; Filiz and Kurt, 2019). The complex interrelations between the plant iron status, the P status, defense reactions and hormone signaling make the promotion of plant nutrition via plant breeding a difficult task.

### Plant-Microbe Interactions Mediating Iron Uptake and Homeostasis

#### Impact of Rhizosphere Microbiota on Iron Availability

The rhizosphere microbiota impacts the physico-chemical properties of the root environment by acidifying the soil through the release of organic acids and protons, and chelating iron with organic acids and siderophores (Figure 1B➊, ➋). These modifications prompt iron extraction from the soil matrix and thus modify its solubility and availability for the host plant.

Solubilization of iron in the rhizosphere is promoted by acidification. Protons are released during microbial activities such as nitrification (Kuypers et al., 2018). Protons may also be released from carboxylic groups when the pH of the soil solution is higher than the pKa of organic acids exuded by microorganisms (Glasauer et al., 2003). The protons released by microbial and plant activities acidify the rhizosphere (Hinsinger et al., 2003; Norton and Ouyang, 2019). Iron initially bound in scarcely soluble minerals (e.g., hematites, goethites) and amorphous solids [e.g., Fe(OH)_3_] is replaced by protons at the sorption sites and released in the soil solution (Figure 1B➊; Albrecht-Gary and Crumbliss, 1998; Glasauer et al., 2003). Acidification also results from phosphate solubilization (Sharma A. et al., 2013). Organic acids are produced by rhizosphere bacteria that solubilize phosphates (e.g., *Pseudomonas*, *Bacillus*, *Rhizobium*, and *Enterobacter*) (Werra et al., 2009; Adeleke et al., 2017). Fe and P are often sequestered in soils together in low-solubility minerals like strengite or phosphosiderite. Thus, increased solubility of iron is associated with increased solubility of P (Marschner et al., 2011; Rijavec and Lapanje, 2016).

Solubilization of iron in the rhizosphere is also promoted by its chelation with organic acids, and by siderophores that scavenge ferric iron immobilized in scarcely soluble or insoluble forms and make it available to plants (Figure 1B➋; Kraemer, 2004; Jin et al., 2010; Ferret et al., 2014). The great majority of aerobic microorganisms synthesize small molecules with a high affinity for ferric iron—called siderophores—for their nutrition in iron stress conditions. Microbial siderophores (MSs) present high but variable affinity for Fe^3+^, and are also diverse in size and chemical composition (Budzikiewicz, 2004; Hider and Kong, 2010; Saha et al., 2016; Khan et al., 2019). It has long been known that plants use iron chelated to MSs for their nutrition in Fe-limiting conditions; in particular, pyoverdines (pvds), a major class of siderophores produced by fluorescent pseudomonads, show a high affinity for ferric iron (reviewed by Crowley, 2006; Vansuyt et al., 2007; Jin et al., 2010; Shirley et al., 2011; Nagata et al., 2013; Radzki et al., 2013; Trapet et al., 2016). Rhizoferrin, ferrocrocin, fusigen, and coprogen, all produced by fungal root symbionts, also display high affinity for ferric iron (Winkelmann, 2017; Haselwandter et al., 2020). Microorganisms are expected to be highly competitive for Fe compared to plant roots because they can (i) use Fe bound to phytosiderophores (PSs) (microbial siderophores like pyoverdine have much higher affinity for Fe than PSs do), (ii) decompose PSs, and (iii) acquire iron more efficiently (Figure 1B⓫; Marschner et al., 2011; Sadrarhami et al., 2021). However, while Fe-pvds are more stable than Fe-PSs, they do not depress plant iron nutrition but, even more, promote it (Vansuyt et al., 2007; Jin et al., 2010; Shirley et al., 2011) in contrast with the early report of Becker et al. (1985b). The mechanisms underlying the beneficial effect of microbial siderophores on plant nutrition remain to be elucidated, even if some insights have been given (Vansuyt et al., 2007; Gonzàlez-Guerrero et al., 2016). Organic acids present much lower affinity for iron than siderophores do, but in circumneutral and alkaline environments such as calcareous soils, organic acids may be deprotonated and thus act as metal-complexing agents (Dehner et al., 2010). Hydrogen cyanide (HCN) produced by microorganisms may also contribute to iron mobilization by chelation (Frey et al., 2010; Rijavec and Lapanje, 2016).

Availability of soil nutrients, including Fe, can also be enhanced by increasing the volume of soil explored (Figure 1B➓). This is achieved by root fungal symbionts which greatly extend the scope of the roots through their fine hyphae. Increases in plant iron content have been ascribed to a better access to soil nutrients via fungal networks (Caris et al., 1998; Liu et al., 2000; Farzaneh et al., 2011). In addition, iron is transported into root cells by endosymbionts (Gonzàlez-Guerrero et al., 2016).

#### Plant Iron Physiology Modulation by the Rhizosphere Microbiota

Rhizosphere microorganisms modulate plant iron uptake mechanisms. The expression of genes involved in strategies I and II is modulated in the presence of microorganisms (Figure 1B➏; Zhang et al., 2009; Nagata et al., 2013; Nagata, 2017; Kobae et al., 2014; Zhou et al., 2016, 2018; Wang et al., 2017; Kabir et al., 2020). Rhizosphere acidification and ferric reductase activity (implied in strategy I) and plant PS synthesis (implied in strategy II) are enhanced (Reza, 2017; Wang et al., 2017; Zhou et al., 2018; Prity et al., 2020). The production of plant phenolics known to impact plant iron uptake and remobilization (Figure 1B➎; Fourcroy et al., 2016; Curie and Mari, 2017; Waters et al., 2018) and iron assimilability in food due to their antinutrient properties (Delimont et al., 2017) is also modified. Fluorescent pseudomonads induce the ISR (Van Loon et al., 2008; Berendsen et al., 2015) which regulates the expression of the root-specific transcription factor MYB72 and the MYB72-controlled beta-glucosidase BGLU42 (Zamioudis et al., 2014). These factors control the synthesis and excretion of iron-mobilizing FPCs in *Arabidopsis* (Palmer et al., 2013). Coumarins improve plant performance by eliciting microbe-assisted iron nutrition (Harbort et al., 2020). The concentration and composition of phenolic compounds in edible parts of plants is regulated by associated microorganisms (e.g., Basha et al., 2006; Lavania et al., 2006; Baslam et al., 2011; Jain et al., 2014; Singh et al., 2014; Baker et al., 2015).

Microorganisms modulate plant hormone signaling, which in turn impacts the plant iron physiology by modulating iron acquisition and homeostasis (Figure 1B➑; Lei et al., 2014; Li et al., 2015; Filiz and Kurt, 2019). Plant hormone signaling is under the control of microorganisms through the elicitation of the induced systemic response (ISR), a plant response interrelated with the plant iron deficiency response (Zamioudis et al., 2014; Romera et al., 2019). Changes in the plant indole acetic acid (IAA) and iron contents are observed concomitantly after bacterial inoculation, suggesting that microorganisms impact together hormone signaling and iron nutrition (Zhou et al., 2016, 2018; Housh et al., 2021). Emission of volatile organic compounds (VOCs) by *Bacillus amyloliquefaciens* promotes plant iron nutrition in *Arabidopsis*, and this promotion requires nitric oxide (NO) regulation (Wang et al., 2017), suggesting that the beneficial effect of the rhizosphere bacterial strain is regulated by plant hormone signaling. Rhizosphere microorganisms may either synthesize or degrade phytohormones [i.e., abscisic acid (ABA); IAA; gibberellic acid (GA); cytokinins (CKs); salicylic acid (SA); ethylene (Et); NO] and therefore modulate phytohormone concentrations (Horchani et al., 2011; Bakker et al., 2014; Egamberdieva et al., 2017; Ravanbakhsh et al., 2018). For example, the concentration of ethylene, a key regulator of root apoplastic iron remobilization under Fe shortage (Curie and Mari, 2017), is regulated by microbial 1-aminocyclopropane-1 carboxylic acid (ACC) deaminase that degrades the Et precursor (Ravanbakhsh et al., 2018).

The influence of microorganisms on the plant antioxidant defense has been associated to an increase of the plant iron content in a series of studies performed on sorghum, sunflower and alfalfa (Figure 1B➐; Kabir et al., 2020; Prity et al., 2020; Rahman et al., 2020). According to these authors, the promotion of ROS-scavenging activities by arbuscular mycorrhizal fungi (AMF) is part of the mechanisms involved in alleviation of Fe-deficiency symptoms.

Rhizosphere microorganisms can modify the plant iron status *via* their influence on the plant P status (Figure 1B➒; Tsai and Schmidt, 2017; Shi et al., 2018; Filiz and Kurt, 2019). Phosphate solubilizers and AMF have long been described to promote plant P nutrition and growth (Brown, 1974; Smith et al., 2011). More recently, microbial promotion of P nutrition was showed to impact iron partitioning in the roots and shoots of *Thypha angustifolia* (Ghosh et al., 2014), and to enhance iron nutrition in chicory through an AMF (*Glomus irregulare* syn. *Rhizophagus irregularis*; Labidi et al., 2012).

Other activities of rhizosphere microorganisms may also influence iron storage in the root apoplast and its remobilization. The hemicellulose composition of the root cell wall influences the amount of stored Fe, while putrescine, a diamine excreted by the roots, is involved in the iron remobilization process (Figure 1; Zhu et al., 2016; Shi et al., 2018). Since cellulose and hemicellulose are degraded by microbial activities, iron storage in the root apoplast is likely to be impacted by the corresponding microorganisms (Figure 1B➍; Lasa et al., 2019). Metatranscriptomic data indicate that the proportion of cellulose degraders is increased in the rhizosphere of cereals (Turner et al., 2013). Also, putrescine is one of the most commonly used substrate by wheat rhizosphere microorganisms (Gała̧zka et al., 2019): microbial degradation of this diamine (e.g., by pseudomonads) is thus likely to regulate plant remobilization of apoplastic iron (Kuiper et al., 2001; Song et al., 2015; Liu et al., 2018).

**FIGURE 2:**
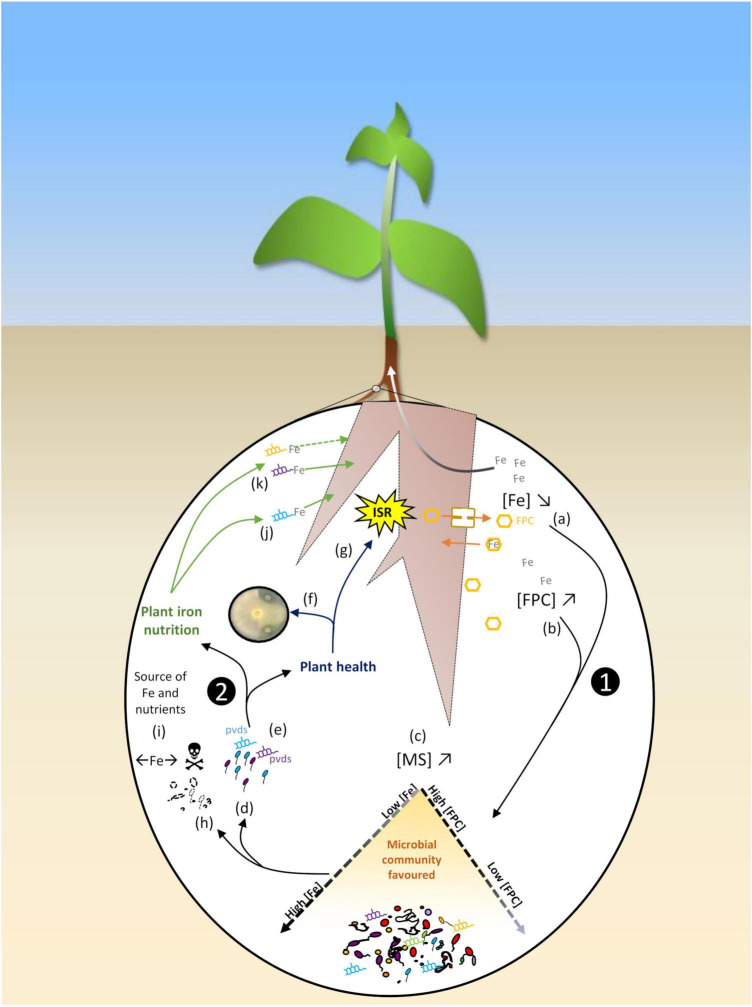
Schematic representation of the feedback loop summarizing the iron dynamics in the rhizosphere as regulated by plant-microbe interactions. ➊The plant iron status shapes the rhizosphere microbiota. Iron bioavailability ([Fe]↘) is decreased in the rhizosphere due to plant Fe uptake (a) (Robin et al., 2006, 2007), and plant excretion of root fluorescent phenolic compounds (FPCs) is enhanced in calcareous soils ([FPC]↗), with low iron availability ([Fe]↘) (b) (Jin et al., 2010; Stringlis et al., 2018). Production of microbial siderophores is consequently increased ([MS]↗) (c) (Jin et al., 2010), and pseudomonads adapted to iron stresss conditions are favored (d) (Robin et al., 2007) thanks to the synthesis of specific pyoverdines (pvds) (e) (Robin et al., 2007; Stringlis et al., 2018). ➋In return, these populations positively influence plant health and iron nutrition. Specific pvds of rhizosphere pseudomonads display high antagonistic activities by competing against phytopathogens for iron (f) (Robin et al., 2007; Gu et al., 2020), and others trigger plant induced systemic resistance (ISR) (g) (Stringlis et al., 2018). Fungal phytopathogens (*Fusarium*) can be counter-selected by FPCs (h) (Stringlis et al., 2018). Specific microbial populations are counter-selected by iron competition (←Fe→), by FPC toxicity (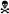), or microbial antagonism; these populations represent a source of iron (and of other nutriments) when metabolized (i). A siderophore produced by a pseudomonad strain recruited in the rhizosphere of an iron-stressed plant can also favor plant iron nutrition (j) (Jin et al., 2010), and distinct pvds of different strains of *Pseudomonas* differently favor plant iron nutrition (k) (Lurthy et al., 2020), suggesting that plant iron nutrition is impacted differently depending on the pseudomonads recruited in the rhizosphere.

In sum, MSs play a key role in plant physiology related to iron uptake and homeostasis (Table 1 and Figures 1, 2) through (i) phosphorus solubilization and thus the plant P status (Sharma A. et al., 2013), (ii) elicitation of plant defense reactions through Microbial Associated Molecular Patterns (MAMPs) inducing ISR (De Vleesschauwer et al., 2006; Höfte and Bakker, 2007; Van Loon et al., 2008), (iii) plant hormone signaling and the synthesis of fluorescent root phenolics via ISR (Pieterse et al., 2014; Zamioudis et al., 2014), and (iv) the expression of genes mediating iron uptake and homeostasis (Table 1). The importance of microbial siderophores in the rhizosphere is also evidenced by results showing that their synthesis and activities are enhanced in the rhizosphere. Protein families related to siderophore production increased in barley root- and rhizosphere-associated bacterial taxa (Bulgarelli et al., 2015), and sequences encoding bacterial siderophore synthesis were highly enriched within bacterial endophytes in rice roots (Sessitsch et al., 2012).

In addition, discussion is running on the possible contribution of microbial siderophores to the remobilization of root apoplastic iron and in a non-reductive process of iron uptake by plants (Figure 1B➌). A large-scale transcriptomic study in *Arabidopsis* suggests that remobilization of root apoplastic iron is promoted by the pyoverdine synthesized by *P. fluorescen*s strain C7R12 (Trapet et al., 2016). Plants grown in iron-deficient conditions in the presence of apo-pyoverdine (a siderophore uncomplexed with iron) exhibited a phenotype similar to that of plants grown in iron-containing medium and incorporated more iron than the untreated plants did. In these conditions, pyoverdine repressed the expression of root genes related to ABA signaling (Trapet et al., 2016), suggesting that the MS may promote the remobilization of root apoplastic iron (which indeed implies ABA regulation) (Lei et al., 2014; Curie and Mari, 2017). Possible non-reductive uptake of bacterial ferrisiderophores would represent an additional and major influence of microorganisms on the plant iron status. Various findings support the existence of a transport system whereby the plant would internalize bacterial ferrisiderophores in the form of integral ferric chelates. Pyoverdines of fluorescent pseudomonads chelated to iron (Fe^3+^-pvd) contribute to the plant iron nutrition of both dicots (strategy I) and graminaceous monocots (strategy II) more efficiently than the synthetic ferric chelate Fe^3+^-EDTA does (Vansuyt et al., 2007; Jin et al., 2010; Shirley et al., 2011). The stability constant of the Fe^3+^-pvd complex is significantly higher (10^32^) than that of Fe^3+^-EDTA (10^25^) or Fe-PS (10^18^) (Vansuyt et al., 2007; Shirley et al., 2011). This suggests that dissociation and ligand exchange between Fe^3+^-pvd and Fe^3+^-PS might not be the sole process accounting for the enhanced iron nutrition by Fe-MS and suggests incorporation of Fe^3+^-pvd by the roots. The presence of pvd *in planta* was confirmed by measurements of ^15^N-labeled Fe^3+^-pvd and by immunodetection using anti-pyoverdine antibodies (Vansuyt et al., 2007; Trapet et al., 2016). The use of *IRT*1 knock-out mutants indicated that this membrane transporter of Fe^2+^ (IRT1) is not involved in iron uptake from Fe^3+^-pvd in strategy I plants (Vansuyt et al., 2007). Thus, Fe-pvd clearly contributes to plant iron nutrition, with evidence of the presence of pvd *in planta* but no proof of the direct uptake of the entire Fe^3+^-pvd complex. The description of a non-reductive iron uptake system in a phytoplankton organism supports a possible incorporation of bacterial ferrisiderophores kept throughout evolution (Kazamia et al., 2018). This hypothesis is also supported by the presence of vesicles in the roots of *Arabidopsis* supplemented with Fe-pvd (Lemanceau et al., 2009); these vesicles mediate the internalization of ferrisiderophores by endocytosis in diatoms (Kazamia et al., 2018).

### Plant-Plant-Microbe Interactions Mediating Plant Iron Nutrition and Homeostasis

The plant iron status is modulated by plant-plant interactions that also involve microbial interactions in non-sterile growing conditions. Intercropping, in which at least two plant species are grown together in the same field, is proposed as a means to increase crop yield and quality in low-input agricultural systems by valuing beneficial plant-plant interactions. Several studies reviewed by Xue et al. (2016) and Dai et al. (2019) reported increased tolerance to IDC of legumes and non-legume dicots in alkaline conditions when they were associated with a cereal in intercropping systems. Intercropping can also modulate plant iron distribution (Xue et al., 2016). Intercropping with grass was even more efficient than adding iron chelates on the yields of blueberries cultivated in sub-alkaline soil (Michel et al., 2019). An increased iron content of peanut grains (1.43-fold) was recorded when peanut was intercropped with maize in calcareous soil (Zuo and Zhang, 2009). Intercropping with oat was as good as in-furrow amendment with chemical Fe chelate (FeDDHA) for alleviating soybean IDC on calcareous soils (Kaiser et al., 2014). However, variations were observed depending on environmental conditions, and Fe amendment was sometimes more reliable. Better knowledge of the biotic interactions involved is therefore required to increase the reproducibility of the results so as to develop these environmentally friendly cropping systems.

Regarding iron uptake, three mechanisms of facilitation may account for the enhancement of iron nutrition in dicots in the presence of graminaceous crop plants.

The first mechanism would rely on the extraction of iron by chelation with PSs from grasses that would increase iron availability to dicots, as shown when intercropping olive (Cañasveras et al., 2014) and citrus rootstocks (Cesco et al., 2006) with grasses. Intercropping impacted PS production and expression of the *FRO* and *IRT* genes implied in the strategy I iron uptake system. However, this trend lacks consistency across studies (Dai et al., 2019).

A second mechanism would rely on a non-reductive mechanism used by dicots to incorporate Fe-PSs formed with PSs excreted by grasses. Fe-PSs from a strategy II plant (maize) were internalized by a strategy I plant (peanut) (Xiong et al., 2013) via a membrane transporter belonging to the YS/YSL family of Fe-PS transporters (Curie et al., 2000, 2009).

Finally, the third mechanism enhancing iron uptake in dicots intercropped with maize would rely on the remobilization of apoplastic iron by root phenolic compounds. Under Fe deficiency, maize was unable to remobilize its pool of root apoplastic iron, contrary to bean (Bienfait et al., 1985). In addition, Fe-deficient bean plants mobilized iron from the root apoplast of other plants grown in their presence (Bienfait et al., 1985). In alkaline conditions, increased synthesis of root fluorescent phenolics (Waters et al., 2018) could contribute to the mobilization of rhizosphere iron by dicots. Therefore, the non-used root apoplastic iron pool of maize roots could be remobilized by an associated dicotyledonous crop. This could partly account for the better iron nutrition of legumes grown together with maize (Xue et al., 2016; Dai et al., 2019).

The rhizosphere microbiota also contributes to the better efficiency of plant species cultivated together. In cereal-legume intercropping, symbiotic interactions between the legume species and nitrogen-fixing microorganisms decrease competition for soil nitrogen, and the resulting resource partitioning promotes nitrogen nutrition of the cereal. In addition to limited interspecific competition for N acquisition in cereal-legume intercropping, other processes such as soil N enrichment or high N restitution through below-ground legume residues benefit N acquisition by the cereal (Hauggaard-Nielsen et al., 2009; Fustec et al., 2010). More generally, plant-plant interactions impact root exudation, and this affects soil rhizosphere microbiota (Vora et al., 2021) and favors colonization by AMF (Ingraffia et al., 2019). On the other hand, improved mycorrhization increased the Fe content in wheat intercropped with faba bean, but did not increase it in mono-cropped faba bean (Ingraffia et al., 2019). According to these authors, the enhancement of plant Fe uptake modulated by AMF depends on soil physico-chemical properties. The mycorrhizosphere of associated plants, formed by AMF-colonized roots and hyphae, increases microbiota functionalities (Wahbi et al., 2016b). Intercropping impacts the abundance, diversity, activity and co-occurrence network of rhizosphere microbial communities (Li et al., 2016, 2018; Wahbi et al., 2016a; Duchene et al., 2017; Taschen et al., 2017; Gao et al., 2019; Zaeem et al., 2019; Liu et al., 2021; Pivato et al., 2021). This is in agreement with the well-known positive relationship between plant and microbial diversity (Spehn et al., 2000; Carney and Matson, 2005; Qiao et al., 2012; Ahmad et al., 2013). In controlled conditions, the pea-wheat association did not harbor a mixture of the two rhizospheres, but rather a new bacterial community with more Actinobacteria and a decreased abundance of α-Proteobacteria and Acidobacteria (Taschen et al., 2017). In another study in field conditions, bacterial networks were impacted by pea-wheat intercropping, but bacterial diversity and structure were not, suggesting a more complex bacterial network and more complex interactions (Pivato et al., 2021). The observed changes in the microbial community diversity and its increased complexity may account for the beneficial effects observed in intercropping. Compared to maize and peanut cultivated independently, comparable microbial communities have been observed whether the roots were separated or not: *Bacillus*, *Brevibacillus*, and *Paenibacillus* were mainly increased in the rhizosphere of maize, while *Burkholderia*, *Pseudomonas*, and *Rhizobium* were mainly increased in the rhizosphere of peanut. In these conditions, the availability of nutrients (N and P) was increased (Li et al., 2018), even if no correlation was found with the changes observed in the microbial community.

More generally, the higher microbial diversity associated with higher plant diversity results in better plant fitness, resilience to stress (De Vries et al., 2018), and positive effects of intercropping (Sun et al., 2009). Various studies suggesting a better iron nutrition of strategy I plants grown in association with a cereal have been reported (reviewed by Xue et al., 2016; Dai et al., 2019). Despite the well-known impact of intercropping on the rhizosphere microbiota and evidence of the role of microorganisms (e.g., AMF) in enhancing plant nutrition in association, data allowing us to evaluate the role played by the plant microbiota are missing. Additional data on the plant iron content will also be required because up to now the effect of intercropping has been mostly evaluated by visually recording IDC symptoms. Therefore, knowledge integrating plant-plant, plant-microorganism and microbe-microbe interactions is sorely lacking.

## Consequences for the Development of Iron Biofortification Strategies

Microorganisms modulate iron bioavailability nearby and within the roots, as weel as plant iron uptake and homeostasis (Figure 1). Optimizing the biotic interactions that mediate plant iron uptake and homeostasis opens onto stimulating prospects for plant iron biofortification. The importance of microorganisms in plant nutrition including iron nutrition is widely acknowledged, but up to now they have been mainly used as biofertilizers and applied to plants in different formulations containing one or several microorganisms. However beneficial effects of microbial inoculation are often offset by a lack of consistency due to poor survival of the introduced strains (Singh and Prasanna, 2020; French et al., 2021).

Current research is now shifting its focus on the monitoring of rhizosphere microbiota on the basis of increasing knowledge of the plant-microbe feedback. The impact of the rhizosphere microbiota on iron availability and plant iron physiology is part of dynamic processes that are themselves influenced by plant-microbe interactions. Monitoring plant-microbe interactions mediating plant iron nutrition and homeostasis requires to decipher the complexity of the corresponding interactions. It is now well established that plants shape the composition of their microbiota via rhizodeposition including root exudation (Badri et al., 2013; Lemanceau et al., 2017a; Canarini et al., 2019; Jones et al., 2019). In turn, the rhizosphere microbiota impacts plant nutrition, growth and health. This feedback loop is modulated by the plant genotype and by the soil physico-chemical and biological properties (Lemanceau et al., 2017b; Rodriguez et al., 2019). These reciprocal interactions are well illustrated by the iron dynamics in the rhizosphere (Figure 2). Two series of studies report that the Fe-chelating ability of the rhizosphere microbiota is modified by the plant iron status. The first one was conducted on transgenic tobacco deregulated in ferritin, hence hyperaccumulation of iron *in planta* and iron depletion of the corresponding rhizosphere. This depletion resulted in the selection of pseudomonad populations highly adapted to iron-stressed conditions thanks to the synthesis of efficient siderophores (Robin et al., 2006, 2007). The second series was conducted with clover grown in Fe-deficient conditions; this plant synthesized more phenolic compounds, hence the selection of a higher occurrence of siderophore-producing bacteria (Jin et al., 2010). In both cases, the plant contributed to decrease rhizosphere iron availability. This led to an increased level of iron competition that favored the microbial communities most adapted to these iron stress conditions thanks to their siderophores (Figure 2; Robin et al., 2006, 2007; Jin et al., 2007, 2010), while depleting those susceptible to low iron availability. The plant metabolites released in iron stress conditions (e.g., phenolic compounds like scopoletin) may even have a biocidal effect on susceptible populations (Gnonlonfin et al., 2012). Microbial populations recruited by the host plant in turn impact plant nutrition, growth, and health (Figure 2). Thus, plant iron nutrition was promoted by siderophores synthesized by a *Pseudomonas* strain originating from the rhizosphere of Fe-deficient clover (Jin et al., 2010). Also, a siderophore from a pseudomonad strain highly represented in the rhizosphere of a pea cultivar tolerant to IDC significantly improved iron nutrition of this plant (Lurthy et al., 2020). Similarly, two strains (*P. simiae* WCS417 and *P. capeferrum* WCS358) highly tolerant to the antimicrobial effect of root phenolics promoted *Arabidopsis* growth *via* siderophore production (Figure 2; Berendsen et al., 2015; Stringlis et al., 2018). The biomass of the microbes counter-selected by iron competition and phenolics represents a potential pool of iron and other nutrients. Thus, in addition to iron stored in ferritins, vacuoles and the root apoplastic compartment, the root microbiota could be used as an additional level of iron storage by plants. Regarding plant health, major phytopathogens are controlled by iron competition in the rhizosphere. Siderophores with a high affinity for iron and retrieved from the rhizosphere of ferritin-overexpressing transgenic tobacco displayed a higher antagonistic activity against the phytopathogenic oomycete *Pythium aphanidermatum* (Figure 2; Robin et al., 2007). Root FPCs synthesized through a MYB72-dependent pathway selectively inhibited the soil-borne fungal pathogens *Fusarium oxysporum* and *Verticillium dahlia* (Figure 2; Stringlis et al., 2018). In addition, plant protection was promoted by the above mentioned *P. simiae* WCS417 and *P. capeferrum* WCS358, inducers of plant systemic resistance (ISR; Stringlis et al., 2018).

The influence of the crosstalk between the host plant and its associated microbiota on plant iron nutrition (Figure 2) stresses the importance of considering the plant together with its microbiota in biofortification strategies. Progress in the knowledge of the interactions between eukaryotic organisms and their associated microbiota has led to the emergence of the holobiont concept, defined as the host and its associated microbes (Vandenkoornhuyse et al., 2015). Because of the importance of their associated microbiota, in terms of abundance, diversity and beneficial effects for the host plant, plants can no longer be considered as stand-alone entities (Dessaux et al., 2016). According to this concept, the genome interacting with its environment is no more restricted to the plant genome but is extended to that of the holobiont (hologenome) (Theis et al., 2016). Therefore, we propose to consider holobiont genetic resources for improving the plant iron status. Including the plant microbiota and its transmission by seeds in breeding programs has been proposed (Gopal and Gupta, 2016; Wei and Jousset, 2017; Berg and Raaijmakers, 2018). More recently, Wille et al. (2019) presented a comprehensive review of the plant-microbe interactions implied in resistance to root diseases in grain legumes and discussed possible consequences for breeding strategies. They especially proposed to consider the entire plant holobiont in resistance breeding strategies. The same principle should be applied to iron biofortification. In that prospect, plant traits included in breeding programs should comprise traits modulating plant-microbe interactions beneficial for the plant iron status. These traits represent promising new breeding targets. Among them, three types stand out and require special attention (i) the synthesis pathways of fluorescent phenolics and plant defense responses sharing common key components, (ii) plant regulation of iron storage in the root apoplast, and (iii) putrescine synthesis mediating apoplastic iron remobilization. The targeted plant traits should also include those involved in the recruitment of functional microbial genes (Lemanceau et al., 2017b) mediating siderophore production, synthesis, or degradation of specific molecules related to the plant iron physiology (e.g., cellulose, hemicellulose, putrescine, plant hormones). Particular attention should be paid to microbial siderophores because they represent a major contribution of microorganisms to the plant iron status, although the mechanisms involved are not all known yet. Recent results show that effects of Fe-MS on the plant iron status vary depending on plant genotype and MS structure (Lurthy et al., 2020). Therefore, the high level of specificity between the plant and its microbiota should be taken into account. Beyond plant-microorganism interactions, crop biodiversity and plant-plant interactions represent major levers for improving the resistance and resilience of canopies and reducing their dependence on synthetic inputs, to ultimately ensure crop sustainability (Wezel et al., 2014; Dubey et al., 2020). Increasing crop biodiversity relies on the association of plants cultivated in intercropping. The challenge is to find out plant associations and practices that favor processes of ecological facilitation in intecropping. This occurs when the association optimizes the development of both species (e.g., improved resource availability) and minimizes any negative interactions that might occur between the two species (Callaway, 1995). To allow this facilitation process to occur and thus promote the functioning and performance of intercropping, the choices of plant species and cultivars to be grown in association, together with the cropping practices (seeding density and pattern, level of nitrogen fertilization) are key to success (Andersen et al., 2007; Neumann et al., 2007; Bedoussac et al., 2015). When these conditions are met, intercropping allows better nutrition of each associated plant species thanks to the facilitation process (Duchene et al., 2017) and the use of fertilizers can be reduced (Bedoussac and Justes, 2010). Research is ongoing to optimize biotic interactions that promote plant nutrition. Given the impact of the plant species, but also of its genotype, on the rhizosphere microbiota, characterizing the effect of different cultivars of a plant species grown in association on the microbial community is a key step for identifying the best performing cultivars in the association. However, the mechanisms underlying the positive effects of these cropping systems on plant-microbe interactions remain largely untapped, and further studies are required to better understand and exploit the interplay of these biotic interactions.

## Conclusion and Prospects

Iron amounts in soils are above plant needs but are not readily available in most agricultural soils. Consequently, increasing soil iron bioavailability to enhance plant and ultimately human nutrition represents a major challenge. The soil microbiota has a great impact on iron bioavailability in the rhizosphere and on plant iron physiology. This should open avenues for plant iron biofortification strategies that will value these biotic interactions. The entire plant holobiont should be considered in biofortification strategies, and the plant traits included in breeding programs should comprise traits modulating plant-microbe interactions beneficial for the plant iron status. These traits will include the synthesis of root phenolics and the regulation of apoplastic iron storage and remobilization. Plant traits mediating the recruitment of microbial genes involved in the synthesis or degradation of specific molecules related to the plant iron physiology (e.g., cellulose, hemicellulose, putrescine, plant hormones) have to be investigated. Special attention should be paid to the interactions with microbial siderophores, which strongly impact the plant iron status; many of the mechanisms involved still have to be identified. Recent findings highlight the specificity of biotic interactions, the role of the environment, and the interconnexion between plant iron nutrition and other parameters that also influence the quantity and the quality of vegetal products—plant health, the P status, and the ionome. This leads us to think that important headways should be made possible by the development of integrative approaches. In addition to iron biofortification, these approaches will take into account plants and their extended genotype formed by each plant and its specific microbiota; this holobiotic organism will be more prone than the plant alone to adapt to environmental stresses. Intercropping appears promising to implement these strategies.

More options for iron biofortification could be brought by emerging research perspectives. Most of the findings on the microbial influence on plant iron come from studies focusing on soil and rhizosphere interactions. Yet, the phyllosphere and spermosphere microbiota, whose influence has long been underestimated, also influence the plant iron physiology (Lemanceau et al., 2017a). Iron is absorbed by the leaves, and a signal originating from the shoots and involving IAA appears to elicit root-to-shoot iron translocation (Kabir et al., 2013; Garnica et al., 2018). The importance of the shoot microbiota on these components of the plant iron dynamics remains to be explored. The spermosphere microbiota is at least partly inherited from parent plants (Lemanceau et al., 2017a). Therefore, it is essential to evaluate the role of the corresponding microorganisms. Studies on the plant microbiota mostly provide taxonomic descriptions of plant-associated microorganisms. Therefore, results are deeply influenced by the soil microbiota reservoir which varies according to the soil physical and chemical properties (Dequiedt et al., 2009; Ranjard et al., 2013; Xue et al., 2018). Lemanceau et al. (2017b) have proposed the principle of a functional plant-genotype-specific core microbiota shared whatever the soil in which the corresponding genotype is cultivated. This proposal relies on the fact that plant-beneficial microbial traits (e.g., production of siderophores, hormones, antibiotic molecules, and HCN) can be found in distinct microbial taxa. Finally, it is important to widen the objectives and develop more integrative studies. Potential trade-offs may indeed occur. Breeding programs focused on plant health promotion could be detrimental to plant growth and also impact the plant iron status, and *vice versa*. The dynamics of iron in the rhizosphere also modulates plant health (Figure 2). Seemingly, increasing plant iron content also more globally impacts the plant ionome (Cohen et al., 1998). This could be favorable to other essential micronutrients like Zn, but could also lead to the accumulation of toxic elements due to the variable specificity of plant iron transporters (Rajkumar et al., 2010; De Valença et al., 2017). Another trade-off probably involves phenolic antioxidant compounds. Research about them is ongoing for improving human health, but they may also act as antinutrients by decreasing iron assimilability. The selection of new genotypes should no longer be oriented toward the production of plants harboring specific characters like enhanced iron content or resistance to a given pathogen. Plant improvement strategies should rather consider the extended genotype formed by the plant and its specific microbiota, and search for combinations allowing the holobiont to quickly adapt to a range of severe biotic and abiotic stresses likely to occur for a particular crop. We should rather tend toward the search for “ideoholotypes.”

## Author Contributions

SM and PL initiated the review. All authors contributed and approved the final manuscript.

## Conflict of Interest

The authors declare that the research was conducted in the absence of any commercial or financial relationships that could be construed as a potential conflict of interest.

## Publisher’s Note

All claims expressed in this article are solely those of the authors and do not necessarily represent those of their affiliated organizations, or those of the publisher, the editors and the reviewers. Any product that may be evaluated in this article, or claim that may be made by its manufacturer, is not guaranteed or endorsed by the publisher.

## Footnotes

1
http://www.ffinetwork.org/
